# Editorial: Challenges and opportunities for the long-term care workforce

**DOI:** 10.3389/fsoc.2026.1976916

**Published:** 2026-09-21

**Authors:** Nadia Brookes, Florin Vadean, Carol Atkinson

**Affiliations:** 1 University of Kent, Canterbury, United Kingdom; 2 Manchester Metropolitan University, Manchester, United Kingdom

**Keywords:** challenges, job quality, long-term care, opportunities, workforce, working conditions

Long-term care (LTC) is under pressure internationally, with rising demand for services and concerns for financial sustainability. As a labor-intensive sector, the supply and quality of care services is crucially dependent on the LTC workforce. However, nearly all countries face challenges in recruiting, deploying, and retaining enough well-trained LTC workers (OECD, 2020). High turnover and attrition rates are mainly attributed to dissatisfaction with working conditions, including low pay, excessive workload, long hours, and poor career prospects (Skills for Care, 2025; Vadean and Saloniki, 2023; Atkinson et al., 2016, 2024). If these issues are not adequately addressed, the current deficits in long-term care work and its quality will lead to an acute and unsustainable global care crisis.

This edited Research Topic highlights some of the challenges and opportunities for the LTC workforce. It comprises of five articles examining issues in England, the United States and Taiwan. The articles cover topics including balancing professionalization with personalization, the impact of staffing issues on the workforce and delivery of care, navigating digital technology in LTC, the importance of emotional labor on retention, and LTC workforce collective bargaining and organizing.

Professionalization of the LTC workforce has been promoted as one way to achieve better pay and employment conditions. Kispeter and Hussein examine the relationship between professionalization and personalization of care and support services in England. Personalization focuses on tailoring care to the individual needs, preferences, and choices of people who draw on care services. Based on qualitative interviews with 25 LTC stakeholders, the findings suggest that these two approaches intersect in the areas of care workers' autonomy, training and registration. Autonomy and skill development should improve care workers' capacity to deliver person-centered services as well as elevate the status of care workers to that of professionals (Atkinson and Crozier, 2020). However, formal training and registration requirements for personal assistants, for example, were perceived by some stakeholders as potentially conflicting with personalization as they may reduce flexibility and care recipients' individual choice. Overall, the study concludes that professionalization and personalization are not necessarily opposing approaches but have a complex relationship.

Research also highlights the link between workforce capacity and quality of care provision. Soto et al. use the example of physical activity (PA) programming in Adult Day Centers (ADCs). Interviews with ADC directors identified staff shortages, limited funding, safety and liability concerns, and the resulting burden on existing staff as key barriers to delivering adequate, evidence-based PA programmes. These workforce challenges may contribute to insufficient or inconsistent PA opportunities that were reflected in participants' relatively poor physical function and elevated risk of falls, morbidity, and mortality. Directors expressed a need for additional support, suggesting that PA specialists, community-based liaisons, and targeted staff training could help build workforce capacity, reduce staff burden and turnover, and improve the delivery of PA programming. Overall, strengthening the workforce appears essential to enabling sustainable, high-quality PA service provision and supporting older adults to maintain independence and quality of life.

Digital technology is often proposed as a solution to revolutionize and support LTC workers to deliver care. Whitfield et al. highlight several workforce issues associated with the adoption of digital technologies in LTC through qualitative case studies with seven care providers. They challenge the assumption that LTC workers limited digital skills are the main barrier, arguing that this is also influenced by the type and design/quality of technology, workers' roles, and the characteristics of care providers. The study emphasizes the importance of supportive learning environments and peer support in helping workers develop and apply digital skills, although limited time and reliance on individual workers can restrict this support. Importantly, poorly designed or malfunctioning digital systems can create additional pressures for LTC workers, who often must use their existing skills and creativity to overcome technological limitations. The authors argue that workforce policy should recognize and value the practical, adaptive, and problem-solving skills of LTC workers rather than focusing solely on providing additional digital training.

A study by Kuo et al. including 365 home care workers in Taiwan looked at the relationship between emotional labor, job satisfaction and willingness to remain in the job. They found that workers who engaged more genuinely with their emotional expressions reported higher job satisfaction and a greater willingness to stay, while job satisfaction itself was not significantly associated with retention intention. These findings highlight the importance of LTC staff abilities to manage the emotional demands of care work. The authors therefore suggest that care organizations must recognize and support the emotional demands placed on workers and to develop effective workforce management and retention strategies.

Workforce challenges in LTC include low pay, insecure employment, poor working conditions, and exploitation, meaning that decent work is rarely available. In England, the quasi-market model of LTC, with services largely outsourced to independent providers, has resulted in a fragmented workforce spread across thousands of organizations, making collective bargaining and trade union activity more difficult (Whitfield, 2022). In Fisher and Foster's study, interviews with LTC workers and those involved in trade union organizing identified four main workforce priorities: improving pay and employment conditions, addressing wider systemic and structural problems, increasing workers' awareness and having their voices heard, and improving workplace environments and practices. Overall, the study argues that employment structures and the low status of care work create significant barriers to improving workers' conditions and strengthening collective organization, highlighting the need for greater recognition, representation, and protection of LTC workers.

The contributors pointed to important challenges faced by the LTC workforce, for example ongoing limitations to care staff autonomy that could improve care workers' capacity to deliver person-centered care as well as their status. The impact of a lack of workforce capacity on staff burden and turnover and the quality of care was also present as well inherent issues with the technology meant to increase care staff productivity. The status of LTC workers and fragmentation of service delivery by independent providers were barriers to collective organizing and efforts to improve working conditions. The research also identified opportunities to strengthen the LTC workforce through training, emotional support, recognition of existing skills and finding a way to make their voices heard.

Together, these five papers reinforce the workforce challenges in LTC and highlight the urgent need for action to improve job quality and working conditions in the sector. In England, at least, there are indications that policy makers maybe recognizing this need, with the Casey Commission's review of how social care is delivered and legislation via the Employment Rights Act (2025) which seeks both to improve working conditions, for example by reducing reliance on unstable employment contracts, and to introduce a Fair Pay Agreement for the LTC sector that will provide a minimum pay floor for care workers. However, it is early days, and it remains to be seen how bold policy makers will be in these efforts and what levels of success will result.
